# A catalogue of the scaleworm genus *Lepidonotus* (Polynoidae, Polychaeta) from South America, with two new records for Brazilian waters

**DOI:** 10.3897/zookeys.533.6184

**Published:** 2015-11-09

**Authors:** José Eriberto De Assis, Rafael Justino de Brito, Martin Lindsey Christoffersen, José Roberto Botelho de Souza

**Affiliations:** 1 Departamento de Zoologia, Centro de Ciências Biológicas - UFPE. Av. Prof. Morais Rego, 1235, Recife, Pernambuco, Brasil. CEP: 50670-901; 2 Departamento de Sistemática e Ecologia, Centro de Ciências Exatas e da Natureza - UFPB, Cidade Universitária, CEP 58059-900, João Pessoa, Paraíba, Brasil

**Keywords:** Benthos, Annelida, catalogue, new record, distribution

## Abstract

The genus *Lepidonotus* is the largest in number of species within the Polynoidae, with more than 70 described species and subspecies. A catalogue of 18 nominal species and subspecies of *Lepidonotus* from South America is provided, with valid names, synonyms and original citations. Redescriptions and illustrations of two species based on new specimens collected along the littoral of the State of Paraíba, northeastern Brazil are included. *Lepidonotus carinulatus* and *Lepidonotus natalensis* are reported for the first time for Brazilian waters. A comparative table of characters for all reported species and subspecies of *Lepidonotus* from South America is provided.

## Introduction

The scaleworm *Lepidonotus* belongs to the family Polynoidae, and contains more than 70 described species (Read and Fauchald 2015). They have been found from the intertidal to the bathyal zones, in diverse marine environments (Day 1967, Fauchald 1977, Salazar-Silva 2006, Wehe 2006).

Leach (1816) established the genus *Lepidonotus* for the species *Aphrodita clava* Montagu (1808), which had been described earlier. This author did not provide identifying characters of the genus, which led to subsequent confusion in the literature, causing many synonyms. Later, Seidler (1923) made a very extensive review of the Lepidonotinae, presenting descriptions and keys to more than 50 species of *Lepidonotus*, but there are almost no illustrations to supplement the descriptions, nor is it clear from the text which specimens or types were examined (Wehe 2006). However, Wehe (2006) clarified that this paper is invaluable in providing base-line data and access to the literature on lepidonotid genera.

*Lepidonotus* has a short body with 26 segments, is dorsoventrally flattened, and subrectangular in the cross-section. The prostomium is bilobed, extending anteriorly into the ceratophores of the terminally-attached lateral antennae. The antennae and cirri are smooth. A facial tubercle is present; the buccal segment is with or without nuchal fold. Twelve pairs of elytra are present on segments 2, 4, 5, 7, 9, 11, 13, 15, 17, 19, 21 and 23; elytra are with or without tubercles and papillae. The notopodia small or vestigial, unidentate notochaetae are short. The neuropodia are large, with or without an acicular lobe; the neurochaetae are stout, long, with subdistal spines and unidentate or occasionally bidentate tips (Fauchald 1977, Amaral and Nonato 1982, Ruff 1995, Wehe 2006).

In this paper, a catalogue of the genus *Lepidonotus* from South America is provided, and *Lepidonotus carinulatus* and *Lepidonotus natalensis* are redescribed, collected in the intertidal region of the State of Paraíba, northeastern Brazil. These two species are reported for the first time for Brazilian waters.

## Material and methods

The species accounts in the catalogue are given alphabetically. Each account contains author, publication year, number of pages, figures, types, and deposition numbers, together with the abbreviation of the museum or institution in which the type material is deposited, type locality with coordinatesand geographical distribution, when available. In some cases, remarks on taxonomic status of somes species are included. Synonyms are listed chronologically. A comparative table for all reported species and subspecies from South American is provided (Table 1).

**Table 1.** Comparative table for all species and subspecies of the genus *Lepidonotus* from South America.

	Characters							
Species	Prostomium	Palps	Nuchal nodules	Posterior part of the elytra	Microtubercule	Macrotubercule	Notochaetae	Neurochaetae
*Lepidonotus arenosus*	Rounded	Smooth	Present	Numerous posterior papillae	Small papillae egg-shaped	Bigger central papillae egg-shaped	Thin capillaries in all chaetigers	Stout, curved, with spinous row, tip bidentate
*Lepidonotus brasiliensis*	Rounded	Smooth	Absent	Numerous thin papillae	Small papillae lunar crate-shaped	Big papillae mushroom-shaped	Spinous ciliated capillaries	Stout, curved, with spinous rows, tip unidentate
*Lepidonotus brasiliensis laevis*	Rounded	Smooth	Absent	Numerous papillae in posterior elytra	Small papillae lunar crate-shaped	Big papillae mushroom-shaped	Thin capillaries, with 3 ciliated filaments	Stout, straight, smooth blade, unidentate
*Lepidonotus caeruleus*	Longer than wide	Papillate	Absent	Numerous short papillae on posterior portion	Conical	Absent	Longer capillaries shorter, curved and dentate; shorter capillaries long and barbed	Long, strongly curved,with spinous row, tip bidentate
*Lepidonotus carinulatus*	Rounded to hexagonal, buccal segment with two nuchal nodules	With 8 rows of longitudi nal papillae	Present	Papillae along external edge	Small warty tubercles	Carinate tubercles scattered	Spinous, superior row slender, serrate on convex side	Stout, falcate, subdistally thickened, few rows of spines, tip bidentate
*Lepidonotus crosslandi*	Rounded, with posterior nuchal fold	Smooth	Absent	Numerous short posterior papillae	Small and thin tubercles	Absent	Fairly numerous capillaries	Stout, slightly curved tip, with a row of spines, bidentate in two chaetigers
*Lepidonotus crosslandi peruana*	Rounded	Smooth	Present	Numerous short posterior papillae	Small and thin tubercles	Absent	Fairly numerous capillaries	Stout, tip slightly curved, with rows of strong spines, bidentate in all chaetigers
*Lepidonotus furcillatus*	Rounded	With numerous papillae	Absent	Digitiform papillae with central dark spot	Small and conical	Small, nodular	Curved, short, smooth	Stout, weakly ornamented, bidentate
*Lepidonotus hupferi*	Rounded to hexagonal	Smooth	Absent	Fringe covering posterior part of elytra	Rounded, surface rough	Warty and pointed	Capillaries spinous in all chetigers	Stout, spinous rows with fine teeth, bidentate
*Lepidonotus margaritaceus*	Convex, posterior rounded	Smooth	Absent	Slender papillae on posterior border	Small granules	Absent	Short, numerous rows of small spines	Stout, numerous rows of distal spines, unidentate
*Lepidonotus natalensis*	Slightly hexagonal	With 8 rows of Longitud-inal papillae		Long digitiform external papillae and group of infero-central papillae	Crown-like tip or without tip	Crown-like tip in central region	Spinous, superior row slender, tips fine, pointed	Stout, falcate, subdistally slightly thickened, few rows of spines, tip unidentate
*Lepidonotus nesophilus*	Convex, posterior rounded	smooth	Absent	Absent	Hemisferical, spinous	Long spines	Spinous capillaries in all chaetigers	Stout, with spinous rows, bidentate
*Lepidonotus panamensis*	Largely hidden, convex lobe projecting over posterior half	A few papillae		Numerous posterior papillae and tulf of long central papillae	Numerous small spines on centre	Excavate disk, cap covered by many spines	Chaetae shorter, falcate	Long, stout, with curved tip, unidentate
*Lepidonotus savygni*	Rounded	Smooth	Absent	Slender short posterior papillae	Numerous small warty-shaped spines	Warty-shaped	Spinous capillaries in all chaetigers	Long, stout, tip curved, unidentate
*Lepidonotus sublevis*	Slightly longer than wide	Papillate	Absent	Additional posterior papillae	Conical, without areola	Absent	Spinous capillaries in all chaetigers	Stout, few spinous rows, tip slightly hooked
*Lepidonotus teinuetosus*	Oblong, no cephalic peaks	Smooth	Absent	Slender posterior papillae	Blunt or somewhat warty tips	Very small, conical or globular	Slender, numerous rows of small spines and capillary tips tapering	Stouter, falcate, numerous distal spine rows, d mostly unidentate, few spines minutely bidentate
*Lepidonotus tomentosus*	Rounded	Smooth	Absent	Numerous large posterior papillae	Small warty tips	Numerous warts on central elytra	Spinous capillaries in all chaetigers;	Stout, with spinous row, bidentate
*Lepidonotus variabilis*	Rounded	Smooth	Absent	Numerous large posterior papillae	Small, cylindrical	Absent	Spinous capillaries in all chaetigers	Stout, with spinous rows, secondary teeth small, bidentate

Specimens were collected by handpicking during low tides from the intertidal region (0.0–0.2 m) and by snorkeling to a depth of up to 5 meters along the coast of the state of Paraíba. Specimens were fixed in formaldehyde (10% in seawater), and later rinsed with fresh water and transferred to 70% ethanol. General structures were observed with Stereomicroscope Olympus Nikon SMZ800. Chaetae and elytrae were observed with an Olympus BX41 compound microscope. All illustrations were drawn using a camera lucida, and photographs were edited in Photoshop, PhotoScape and CorelDraw X7. Specimens are deposited in the ‘Coleção de Invertebrados Paulo Young’, Departamento de Sistemática e Ecologia da Universidade Federal da Paraíba’, Brazil.

The nomenclature of appendages and other characteristics of polynoids mentioned in this paper follow Tebble and Chambers (1982), Hanley and Burke (1991), Ruff (1995), Imajima (1997), and Wehe (2006).

The following abbreviations are used in the text:

AMNH American Museum of Natural History, New York

BMNH The Natural History Museum, London, Great Britain (formerly British Museum of Natural History)

LIPY Laboratório de Invertebrados Paulo Young, Paraíba, Brazil

MNHN Poly Type
Polychaeta type collection, Museum National d’Histoire Naturelle, Paris

NCB Naturalis, The Netherlands Centre for Biodiversity, Leiden

PMNH Peabody Museum of Natural History, Yale University

POLY-UFPB Coleção de Polychaeta do Laboratório de Invertebrados Paulo Young

SSM, Naturhistoriska Rijsmuseet, Stockholm

USNM National Museum of Natural History, Smithsonian Institution, Washington D.C.

ZUEC-POL Polychaete Collection, Zoological Museum of the State University of Campinas, São Paulo

ZMB Naturhistorisches Forschungsinstitut, Museum für Naturkunde, Zentralinstitutder Humboldt-Universität zu Berlin, Germany

ZMH Zoologisches Institut und Museum der Universität Hamburg, Germany

## Results

### Family Polynoidae Malmgren, 1867

#### Genus Lepidonotus

Taxon classificationAnimaliaPhyllodocidaPolynoidae

Leach, 1816

##### Type species.

*Aphrodita clava* Montagu, 1808

Leach 1816, Hanley and Burke 1991, Ruff 1995, Chambers and Muir 1997, Barnich and Fiege 2003.

##### Diagnosis.

Body short, arched, with 26 segments. Bilobed prostomium extending anteriorly into ceratophores of terminally-attached lateral antennae. Antennae and cirri smooth. Facial tubercle present; buccal segment with or without nuchal fold. Twelve pairs of elytra on segments 2, 4, 5, 7.... 21 and 23. Notopodia small or vestigial; unidentate notochaetae short, slender, spinose, or notochaetae capillaries sometimes present. Neuropodia large, with or without acicular lobe; neurochaetae stout, long, with subdistal spines and unidentate or occasionally bidentate tips.

##### Remarks.

The genus *Lepidonotus* contains more than 70 species distributed worldwide (Ruff 1995). However, only 18 species and subspecies have been reported for South America, including the two new records described here.

#### 1. Lepidonotus arenosus

Taxon classificationAnimaliaPhyllodocidaPolynoidae

Ehlers, 1901b

Lepidonotus arenosus Ehlers, 1901b: 253–254 (Calbuco, Chile), 1901a: 49, pl. 2: figs 9–12 (Chile).-Wesenberg-Lund 1962: 15.-Hartwich 1993: 80.-Pleijel 2007: 179 (New Caledonia).

##### Holotype.

NCB Verm. 3643.

##### Type locality.

Calbuco, Chile.

##### Distribution.

Chile and New Caledonia.

#### 2. Lepidonotus brasiliensis

Taxon classificationAnimaliaPhyllodocidaPolynoidae

(Quatrefages, 1866)

Polynoe brasiliensis Quatrefages, 1866: 246–247 (Bahia, Brazil).-Solís-Weiss et al. 2004: 358.
Lepidonotus brasiliensis -Seidler 1924: 37.-Amaral and Nonato 1982: 25.-Salazar-Vallejo 1996: 15.-Amaral et al. 2013.

##### Syntype of *Polynoe brasilienis*.

MNHN Poly Type 78.

##### Type locality.

Bahia, Brazil.

##### Distribution.

Known only from the type-locality in Bahia.

#### 3. Lepidonotus brasiliensis laevis

Taxon classificationAnimaliaPhyllodocidaPolynoidae

Rullier & Amoureux, 1979

Lepidonotus braziliensis laevis Rullier & Amoureux, 1979: 150, fig. d. (Brazil).-Solís-Weiss et al. 2004: 358.

##### Syntype.

MNHN Poly Type: 1304.

##### Type locality.

Bahia, Brazil.

##### Distribution.

This species occurs along the Brazilian littoral.

##### Remarks.

Solís-Weiss et al. (2006) considered only the species, however, in Read and Fauchald (2015), the status as subspecies is considered valid.

#### 4. Lepidonotus caeruleus

Taxon classificationAnimaliaPhyllodocidaPolynoidae

Kinberg, 1856

**Figure 1.**
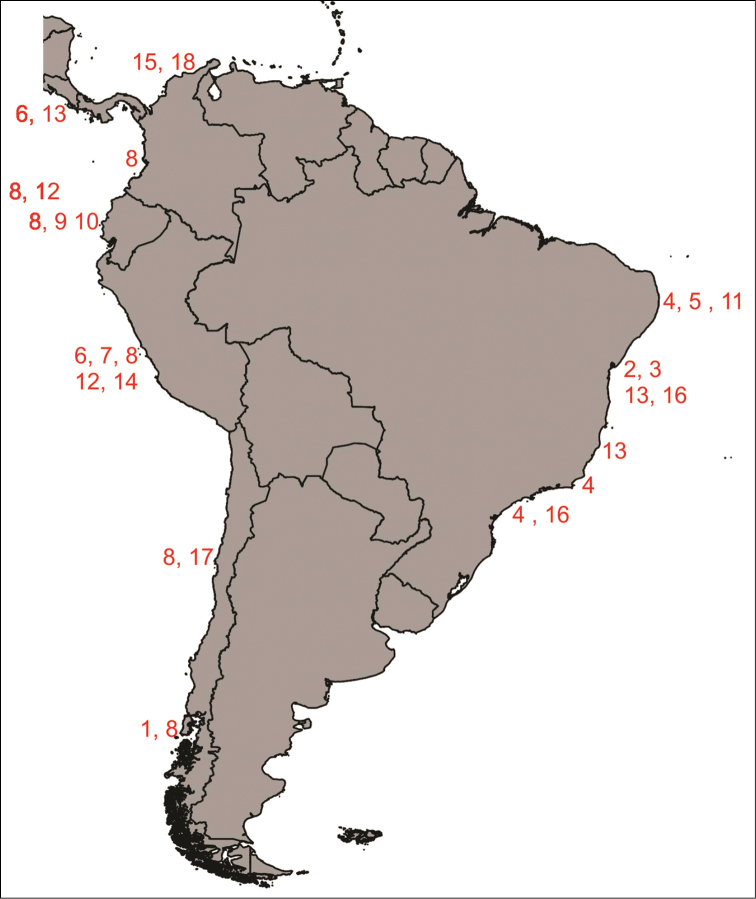
Map showing the distribution of genus *Lepidonotus* in South America: **1** *Lepidonotus arenosus* **2** *Lepidonotus brasiliensis* **3** *Lepidonotus brasiliensis laevis* **4** *Lepidonotus caeruleus* **5** *Lepidonotus carinulatus* **6** *Lepidonotus crosslandi* **7** *Lepidonotus crosslandi peruana* **8** *Lepidonotus furcillatus* **9** *Lepidonotus hupferi* **10** *Lepidonotus margaritaceus* **11** *Lepidonotus natalensis* **12** *Lepidonotus nesophilus* **13** *Lepidonotus panamensis* **14** *Lepidonotus savignyi* **15** *Lepidonotus sublevis* **16** *Lepidonotus tenuisetosus* **17** *Lepidonotus tomentosus* **18** *Lepidonotus viriabilis*.

**Figure 2.**
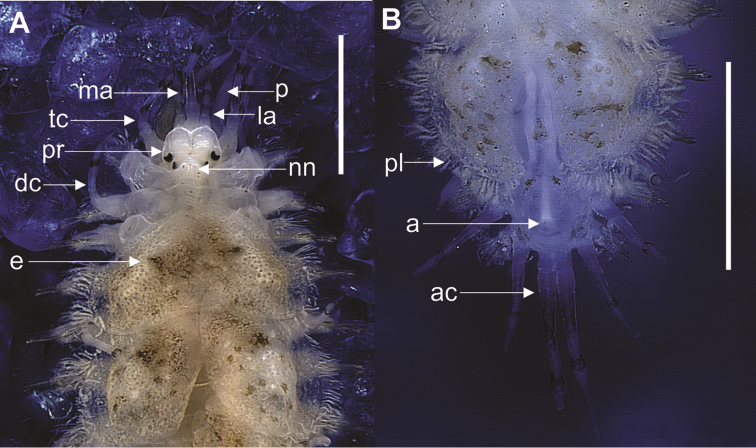
**A** Anterior end of *Lepidonotus carinulatus* showing the dark ring in the antennae and tentacular cirrus (anterodorsal view of the prostomium) **B** Anterior end showing the dorsal anus in the last chaetigerous segment. Scale bars: 1 mm (**A, B**). (p, palp; ma, median antennae; la, lateral antennae; tc, tentacular cirri; pr, prostomium; nn, nuchal nodules; dc. Dorsal cirrus; e, elytra; pl, papillae; a, anus; ac, anal cirri.

**Figure 3.**
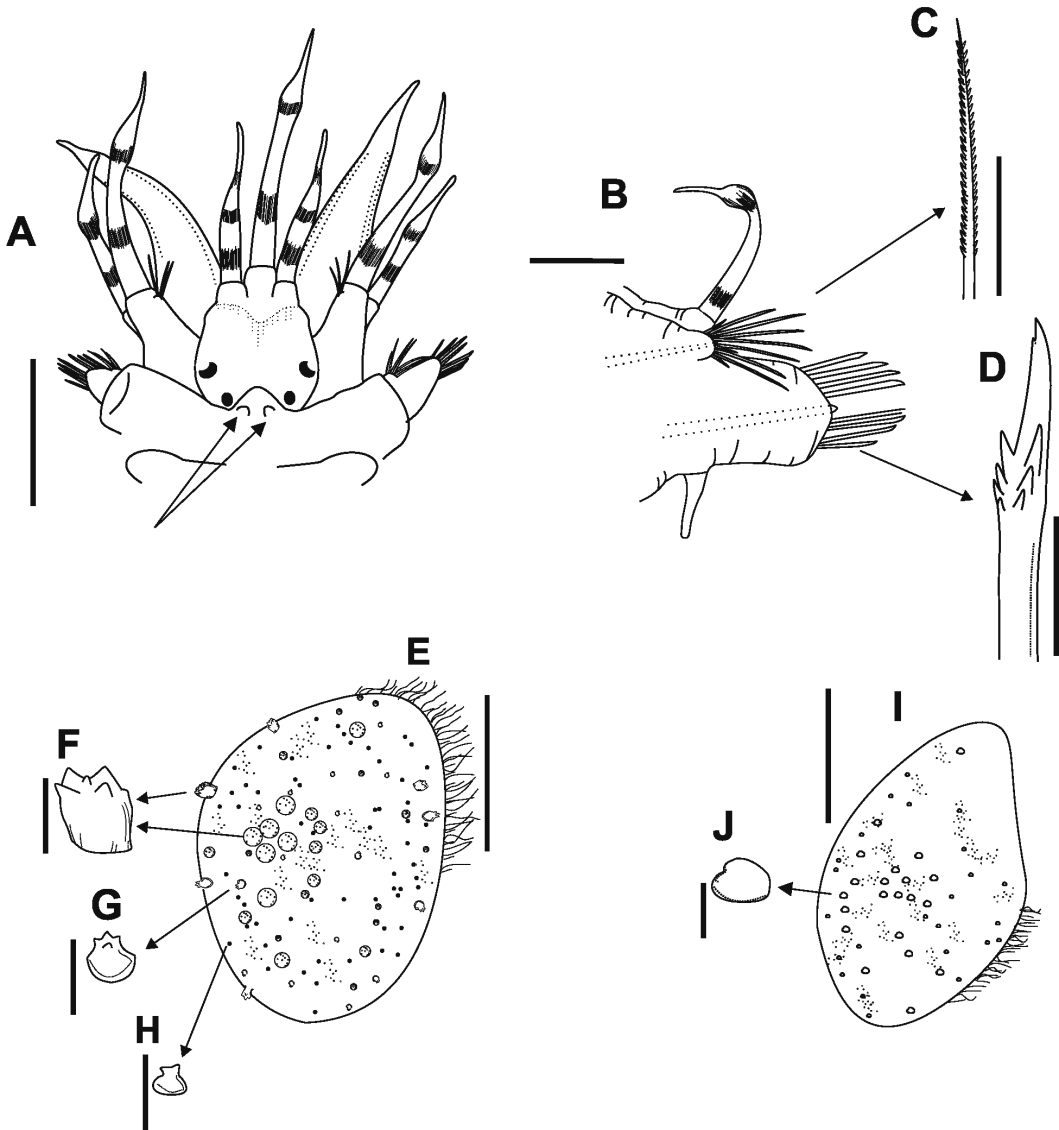
*Lepidonotus carinulatus* **A** pair of nuchal nodules on 2nd segment in dorsal anterior view **B** right parapodia of 4th segment, dorsal view **C** notochaetae, dorsal view **D** neurochaetae, ventral view **E** first right elytra, dorsal view **F–G** macrotubercle **H** microtubercles **I** right elytra of 5th segment **J** macrotubercle. Scale bars: 1 mm (**A**); 0,5 mm (**B–D**), 0.05 mm (**E–J**).

**Figure 4.**
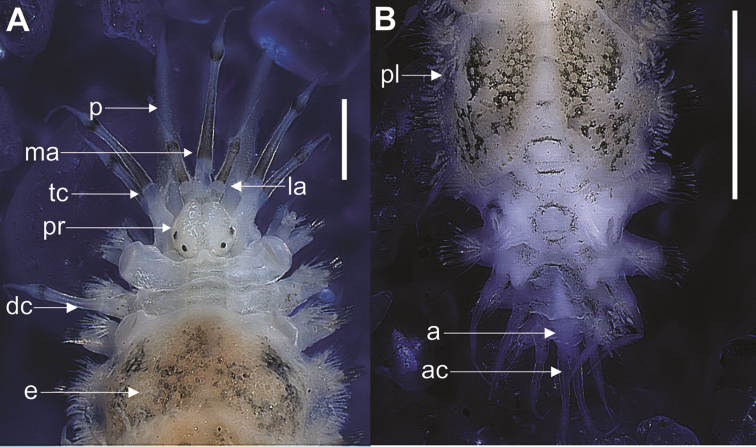
**A** Anterior end of *Lepidonotus natalensis* showing the pigmentation of antennae and tentacular cirri **B** dorsal anus in the 23rd segment chaetigerous, dorsal posterior view. Scale bars: 1 mm (**A–B**). (p, palp; ma, median antennae; la, lateral antennae; tc, tentacular cirri; pr, prostomium; nn, nuchal nodules; dc, dorsal cirrus; e, elytra; pl, papillae; a, anus; ac, anal cirri.

**Figure 5.**
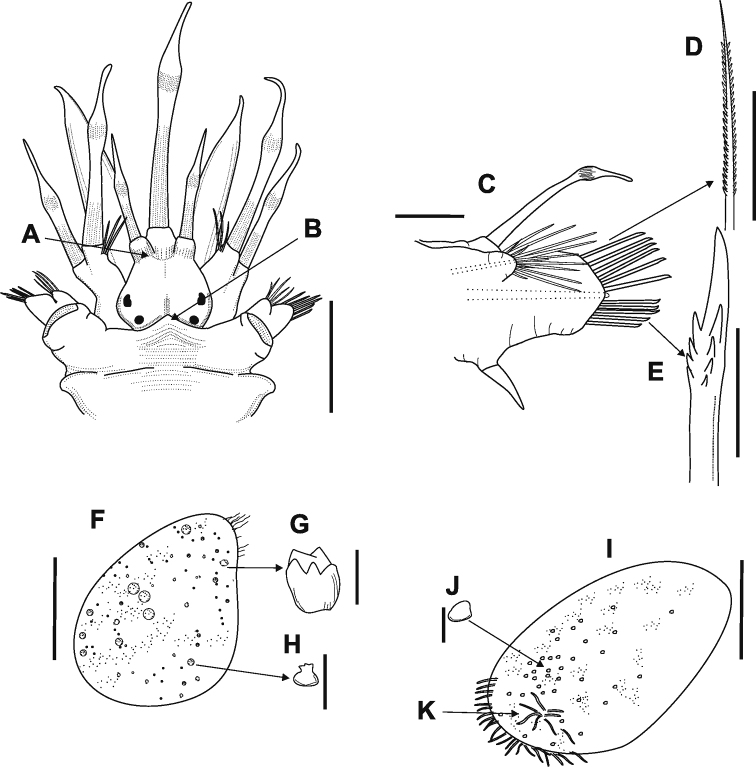
*Lepidonotus natalensis* **A** anterodorsal view of prostomium **B** second segment showing the anterior peak **C** right parapodia of 4th segment, dorsal view **D** notochaetae, dorsal view **E** neurochaetae, ventral view **F** first right elytra, dorsal view **G** macrotubercle **H** microtubercles **I** right elytra of 6th segment **J** macrotubercle **K** group of papillae. Scale bars: 1 mm (**A**); 0,5 mm (**B–D**); 0.05 mm (**B–D**).

Lepidonotus caeruleus Kinberg, 1856: 384 (off Rio de Janeiro-Brazil), 1858: 13–14, pl. 4: fig. 16, pl. 10, fig. 51.-Baird 1865: 183.-Grube 1876: 61.-Seidler 1924: 69.-Hartman 1939: 108–109.-Nonato and Luna 1970a: 63 (Alagoas, 19–35 m); 1970b: 66–67, pl. 1: figs 8–14 (Alagoas, 19–35 m).-Rullier and Amoureux 1979: 152 (Alagoas and Bahia).-Morgado and Amaral 1981: 93 (São Paulo, in bryozoan colonies).-Amaral and Nonato 1982: 25.-Salazar-Vallejo 1996: 15.-Duarte and Nalesso 1996: 142 (São Paulo, in colonies of *Zygomycale parishii*).-Morgado and Tanaka 2001: 178 (São Paulo; in colonies of *Schizoporella errata*).-Berlandi et al. 2012: 282 (off Espírito Santo State, rhodolith beds).-De Assis et al. 2012: 17 (Paraíba).-Cunha et al. 2013: 146 (off Bahia).
Polynoe caerulea .-Quatrefages 1866: 224.
Lepidonotus caeloris .-Moore 1903: 412–414, pl. 23: fig. 12 (Japan, 115–280 m), 1906: 546–547, pl. 36: figs 36–37 (Alaska); 1908: 331 (Alaska and Pacific Canada), 1910: 333–334 (California).-Essenberg 1918: 184 (Alaska to California, 53–932 m).-Hartman and Reish 1950: 5 (Oregon).-Díaz-Castaneda and Rodríguez-Villanueva 1998: 12 (Pacific Mexico).
Polynoë caelora .-Izuka 1912: 23–25, fig. (Japan).
Lepidonotus caelorus .-Treadwell 1914: 182 (California).-Chamberlin 1918: 174 (California).-Berkeley 1923: 213 (Pacific Canada).-Hartman 1939: 44, 1944: 244 (California).-Rioja 1941: 680 (Pacific Mexico), 1947: 199 (Pacific Mexico).-Berkeley and Berkeley 1942 (Alaska).-Pettibone 1953: 15–16, pl. 1: figs 1–8; pl. 2: figs 9–19 (Washington and Oregon 7.3–256 m, with *Volsella modiolus*, *Balanus nubilis*, on tube of *Neosabella* [as *Sabellaria*] *cementarium*, in calcareous tubes of Dodecaceria “pacifica”).-Reish 1968: 100 (California).
Lepidonotus coelorus .-Treadwell 1937: 141 (California).-Berkeley and Berkeley 1942: 187 (Pacific Canada), 1948: 9–10, figs 6–7 (Pacific Canada).-Pequegnat 1964: 278 (California).
Lepidonotus caerulus .-Berkeley and Berkeley 1941: 20 (California).

##### Holotype.

ZUEC-POL 2919.

##### Type locality.

off Rio de Janeiro-Brazil.

##### Distribution.

Western Pacific from Japan. Eastern Pacific from Alaska to California. Southwestern Atlantic from Paraíba to São Paulo. 7.3–932 m (Figure 1).

##### Biology.

The species occurs in colonies of the sponge *Zygomycale parishii* (Bowerbank, 1875), in colonies of the bryozoan *Schizoporella errata* (Waters, 1879), on tubes of sabellariid *Neosabellaria cementarium* (Moore, 1906), in tubes of cirratulid Dodecaceria “pacifica”, with the barnacle *Balanus nubilis* (Darwin, 1854), and the mussel *Volsella modiolus*. In rhodolith beds.

##### Remarks.

*Lepidonotus caeruleus* presents a wide distribution. Futher studies are required to enable us to understand if it is a cryptogenic species, because there are no studies to show that it represents a species complex, and its origin was not determined.

#### 5. Lepidonotus carinulatus

Taxon classificationAnimaliaPhyllodocidaPolynoidae

Grube, 1869

Figures 2 , 3

Polynoe (Lepidonotus) carinulata — Grube 1869; Grube 1878: 26–27, pl. 3: figs 2–2 b.
Lepidonotus carinulatus .—Willey 1905: 248–249, pl. 1: figs 7–11, Fauvel 1911: fig. 1, 1918, 1919a, Augener 1922: figures 3–3b, Seidler 1923, Fauvel 1933, Wesenberg-Lund 1949, Fauvel 1955, Mohammad 1971, Day 1975: figs 2 g–k, Amoureux et al. 1978, Hanley and Burke 1991: fig 20, Imajima 1997: figs 45–46, Rasheed and Mustaquim 2003: figs 7–8, Barnich et al. 2004.
Lepidonotus tenuisetosus . — Mohammad 1971: 288, Gravier 1902.

##### Diagnosis.

With two nuchal nodules and without nuchal folds; black pigmentation on antennae, cirrophores and tentaculophores; elytra with dark pigmentation; elytral surface reticulate, with oval to rounded macro- and microtubercles, anterior ones flattened, smooth or carinate, central and posterolateral ones warty; margin with fringing papillae; neurochaetae bidentate.

##### Description.

Body elongated, flattened dorsoventrally, subrectangular in cross-section; 2 mm in length, including palps and pygidial cirri; 26 chaetigerous segments, and pygidium (Figure 2a–b). Prostomium bilobed, rounded to hexagonal, lateral antenna with terminal insertion (Figure 3a). Two pairs of eyes; anterior pair dorsolateral, near widest portion of prostomium, posterior pair near posterior end of prostomium, converging towards midline, buccal segment without nuchal fold, but with pair of nuchal nodules (Figure 3a–b). Median and lateral antennae, tentacular and dorsal cirri with two dark rings (Figure 2a), both having subdistal swelling, culminating abruptly in sharp point; ceratophores cylindrical, median antenna larger than lateral antennae. Pair of palps, slightly smaller than median antenna, culminating in thin point, with 8 longitudinal rows of papillae.

Tentacular segment with two pairs of cylindrical tentaculophores, with three prostomial chaetae on anterodorsal bases. Buccal cirri larger than ventral cirri, with cylindrical cirrophores. Pharynx with nine pairs of papillae and two pairs of maxillae. Facial tubercle present. Dorsal cirri with same coloration as median antenna, larger than ventral cirri, with cylindrical cirrophores.

Parapodia biramous (Figure 3b), prechaetal lobe quadrate or subtriangular, postchaetal lobe short and subtriangular, both with acicula; short notopodia on anterodorsal side of neuropodia. Notochaetae spinous, superior row slender, serrated on convex side (Figure 3c). Neuropodia large, truncate, distally cleft with prechaetal lobe slightly longer than poschaetal lobe. Neurochaetae stouter than notochaetae, falcate, subdistally thickened, with several rows of spines, distal spines usually much larger than basal ones, and bidentate tips with small secondary tooth (Figure 3d).

Twelve pairs of elytra, covering dorsum entirely, with dark-brown pigmentation; pairs until last chaetiger segment following order: 2, 4, 5, 7, 9, 11, 13, 15, 17, 19, 21 and 23; long papillae along external edge. First three pairs of elytrae more ornate, with tubercles (Figure 3e); on 11th and 12th pair with ornamentation similar to that in first pair. Most carinate macrotubercles in central region (Figure 3f–g), surrounded by microtubercles (Figure 3h); after 4th or 5th pair (Figure 3i), small warty tubercles give impression of smooth elytra (Figure 3j).

Nephridial papillae starting from chaetiger 7, with peduncular aspect. Short ventral cirri with thin tip; pair of long anal cirri with same coloration as median antenna; dorsal anus in last chaetiger segment (Figure 2b).

##### Habitat.

Recorded between the intertidal and 60 m; elsewhere reported down to 200 m (Hartmann-Schröder and Hartmann 1991, Wehe 2006).

##### Syntype.

ZMB 1071.

##### Type locality.

Bohol, Philippines.

##### Distribution.

Red Sea, Arabian Sea: Socotra Archipelago, Gulf of Oman, Arabian Gulf. Elsewhere: Indo-West Pacific: Madagascar, Sri Lanka, Indonesia, Philippines, Hong Kong, South China Sea, Australia, New Caledonia, Japan (Willey 1905, Fauvel 1953, Hanley and Burke 1991, Hanley 1992, Imajima 1997, Barnich et al. 2004).

New records: Coast of Paraíba, Brazil: Pedra da Galé, Pitimbú (07°28'17"S, 34°47'26"W), POLY–UFPB 1501; Rio Mamanguape (06°48'44"S, 34°54'48"W), POLY–UFPB 1502, 1503.

##### Remarks.

Zenetos et al. (2010) assigned *Lepidonotus carinulatus* as an exotic species with an origin in the Indo-Pacific/Red Sea. Its establishment success in the Mediterranean is questionable, because its description, based on local specimens, was insufficient. It is an exotic species in Brazilian waters with casual establishment success; because only the present records are known, it is presumed to be non-established in the Mediterranean area (Zenetos et al. 2010).

#### 6. Lepidonotus crosslandi

Taxon classificationAnimaliaPhyllodocidaPolynoidae

Monro, 1928

Lepidonotus crosslandi Monro, 1928: 553–555, figs 1–4 (Callao and Bahia Independencia, Peru).-Hartman 1939: 109, pl. 5, figs 62–69, 1959.-Fauchald and Reimer 1975: 80 (Panama).-Reimer 1976: 242 (Pacific Panama).-Fauchald 1977: 6 (Pacific Panama).-Von Prahl et al. 1979 (Pacific Colombia).-Cruz et al. 1980: 92 (Ecuador).-Laverde-Castillo 1986 (Colombian Pacific).-Rivera 2008: 23 (El Salvador).

##### Holotype.

USNM 54378.

##### Type locality.

Balboa and Taboga, Panama.

##### Distribution.

El Salvador to Peru.

#### 7. Lepidonotus crosslandi peruana

Taxon classificationAnimaliaPhyllodocidaPolynoidae

Hartmann-Schröder, 1962b

Lepidonotus crosslandi .-Hartman 1939: 42–43, pl. 5: figs 62–69 (Peru, 0–112.8m). [not *Lepidonotus crosslandi*Monro 1928]
Lepidonotus crosslandi peruana Hartmann-Schröder 1962b: 109–112, pl. 1: fig. 3; pl. 2: figs 1–2, 4 (Peru).-Hartman 1965: 9.-Romero et al. 1988: 138 (Peru).

##### Holotype.

ZMH.

##### Type locality.

Callao and Bahia de Independencia, Peru

##### Distribution.

Presently known only from Peru. 0–112.8 m.

#### 8. Lepidonotus furcillatus

Taxon classificationAnimaliaPhyllodocidaPolynoidae

Ehlers, 1901b

Lepidonotus furcillatus Ehlers, 1901b: 254–255 (Tumbes and Cavancha, Chile), 1901a: 52–53, pl. 2, figs 1–8 (Chile).-Augener 1913: 102–103 (Australia).-Seidler 1924: 67–69.-Hartman 1939: 42, pl. 5: figs 57–58 (Colombian Pacific, Ecuador, Pacific Panama, and Chatham Island, New Zealand, 55–101m).-Wesenberg-Lund 1962: 15.-Day 1975: 171 (Australia).-Laverde-Castillo 1986.-Blake 1991 (Galapagos).-Hartwich 1993: 101.-Salazar-Vallejo and Londoño-Mesa 2004: 49.

##### Syntypes.

NCB 3682, 3701.

##### Type locality.

Tumbes and Cavancha, Chile.

##### Distribution.

Western Pacific from Australia and New Zealand. East Pacific from Colombia to Chile and Galapagos Islands.

#### 9. Lepidonotus hupferi

Taxon classificationAnimaliaPhyllodocidaPolynoidae

Augener, 1918

Lepidonotus hupferi Augener, 1918: 133–136, pl. 2: figs 7–11 (Gold Coast, western Africa, Gana).-Seidler 1924: 69–70.-Day 1934: 20 (Angola).-Hartman 1939: 43, pl. 6: figs 78–82 (Ecuador, Pacific Panama, and Pacific Mexico, 3.7–22 m).-Steinbeck and Rickets 1941 (Pacific Mexico).-Rioja 1947: 198–199, figs 1–8 (Pacific Mexico), 1962: 141 (Pacific Mexico).-Tebble 1955: 80 (Australia).-Fauvel and Rullier 1957: 48 (Senegal), 1959a: 146 (Gulf of Guinea), 1959b: 500 (Senegal and Cape Verde).-Day 1967: 37 (southern Africa).-Intes and Le Loeuff 1975: 275 (Ivory Coast, 10–30 m).-Cruz et al. 1980: 92 (Ecuador).-Batisda-Zavala 1993 (Pacific Mexico).-Hernández-Alcantara et al. 2003: 6 (Pacific Mexico).-Salazar-Vallejo and Londoño-Mesa 2004: 49.-Pleijel 2007: 179 (New Caledonia).

##### Holotype

*Lepidonotus hupferi*. Fauvel, 1950: 345 (Senegal). ZMH V-530

##### Type locality.

Gold Coast, western Africa, Ghana.

##### Distribution.

Eastern Atlantic from Senegal and Cape Verde. Western Pacific from New Caledonia and Australia. Eastern Pacific from Mexico to Ecuador. 10–30 m.

##### Remarks.

*Lepidonotus hupferi* presents a wide distribution. Futher studies are required to enable us to understand if it is a cryptogenic species, because there are no studies to show that it is a species complex, and its origin was not determined

#### 10. Lepidonotus margaritaceus

Taxon classificationAnimaliaPhyllodocidaPolynoidae

Kinberg, 1856

Lepidonotus margaritaceus Kinberg, 1856: 383 (Guayaquil, Ecuador); 1858: 11–12, pl. 3: fig. 12; pl. 10: fig. 49.-Baird 1865: 182.-Grube 1876: 62.-Seidler 1924: 33–34.-Hartman 1948: 23, pl. 3: figs 1–3.
Polynoe margaritacea .-Quatrefages 1866: 223–224.

##### Holotype.

SSM.

##### Type locality.

Guayaquil, Ecuador.

##### Distribution.

Known only from the type material from Ecuador.

#### 11. Lepidonotus natalensis

Taxon classificationAnimaliaPhyllodocidaPolynoidae

Day, 1951

Figures 4 , 5

Lepidonotus natalensis Day, 1951; fig. 1 e–l [removed from synonymy with *Lepidonotus tenuisetosus* (sensu Day 1967)].
Lepidonotus tenuisetosus Fauvel, 1927: 414 [not Gravier, 1902].

##### Diagnosis.

Without nuchal fold; some elytrae with group of papillae in center, dark pigmentation and small tubercles after 4th or 5th pair, giving impression of smooth elytra; elytra margin and surface with long slender, digitiform papillae; notochaetae partially threadlike; neurochaetae unidentate.

##### Description.

Body elongate, flattened dorsoventrally; with 26 chaetigerous segments, 2 mm in length, including palps and pygidial cirrus, 2 mm in width, including chaetae (Figure 4a–b). Dorsum and sides of parapodia pigmented black. Prostomium bilobed, rounded, slightly hexagonal, black pigmentation at base of ceratophores (Figure 5a). Lateral antenna with terminal insertion; two pairs of eyes, anterior pair dorsolateral, near widest portion of prostomium, posterior pair near base of prostomium, converging towards midline (Figure 5b). Median and lateral antennae, tentacular and dorsal cirri with two dark rings; first more elongated than second, both having subdistal swelling, culminating abruptly in sharp point; ceratophores cylindrical, median antenna larger than lateral antennae. Palps paired, same length as median antenna, culminating almost abruptly into thin point, with 8 longitudinal rows of papillae.

Tentacular segment with two pairs of cylindrical tentaculophores, with three prostomial chaetae on anterodorsal bases. Buccal cirri larger than ventral cirri, with cylindrical cirrophores. Pharynx with 9 pairs of papillae and 2 pairs of maxillae. Facial tubercle present. Dorsal cirri with same coloration as median antenna, larger than ventral cirri, with cylindrical cirrophores.

Parapodia biramous (Figure 5c), prechaetal lobe quadrate or subtriangular, postchaetal lobe short and subtriangular, both with acicula; short notopodia on anterodorsal side of neuropodia. Notochaetae spinous, superior row slender, partially thread-like, with fine, pointed tips (Figure 5d). Neuropodia large, truncate, distally cleft, with prechaetal lobe slightly longer than poschaetal lobe. Neurochaetae stouter than notochaetae, falcate, subdistally slightly thickened, with few rows of spines below slightly bent, blunt, unidentate tips (Figure 5e).

Twelve pairs of elytra covering dorsum entirely, with dark or dark-brown pigmentation; pairs until last chaetiger segment following order: 2, 4, 5, 7, 9, 11, 13, 15, 17, 19, 21 and 23. First three pairs more ornate, with macro and microtubercles (Figure 5f-h); these tubercles located more posteriorly on elytra after 4th or 5th pair, giving impression of smooth elytra; on 11th and 12th pairs ornamentation similar to that in first pairs. Most of macrotubercles on central region, surrounded by microtubercles (Figure 5i-j). Very long digitiform papillae along external edge, and group of papillae near inferior base of elytra or sometimes next to center (Figure 5k).

Nephridial papillae starting from chaetiger 7, with peduncular aspect. Short ventral cirri with thin tip; one pair of short anal cirri with same coloration as median antenna; anus dorsal in 23rd chaetigerous segment (Figure 4b).

##### Habitat.

Intertidal zone (algae, rhodoliths) to 5 m, from the coast of Paraíba.

##### Holotype.

BMNH 1961.16.1

##### Type locality.

Natal, South Africa.

##### Distribution.

Suez Canal, Arabian Sea: Karachi; Port Edward, Natal, South Africa, Indian Ocean (Day 1951; Wehe 2006).

##### New records.

Barra de Camaratuba, Mataraca, Paraíba, Brazil (06°36'14.17"S, 34°57'48.79"W).

(POLY-UFPB 1504), adult from Barra de Camaratuba, Mataraca (06°36'14.17"S, 34°57'48.79"W), Paratypes (POLY-UFPB 1505, 1506), Prainha, Baía da Traição (06°41'23.77"S, 34°55'48.47"W), Paraíba, Brazil.

##### Remarks.

*Lepidonotus natalensis* presents a wide distribution. Futher studies are required to enable us to understand if it is a cryptogenic species, because there are no studies to show that it is a species complex, and its origin was not determined.

#### 12. Lepidonotus nesophilus

Taxon classificationAnimaliaPhyllodocidaPolynoidae

Chamberlin, 1919

Lepidonotus nesophilus Chamberlin, 1919: 75–78, pl. 4: figs 1–7; pl. 5: fig. 13 (Chatham Island, Galapagos Archipelago).-Hartman 1939: 38–39, pl. 7: figs 83–95 (Galapagos Islands, 22–73 m) [not Tenacatitla Bay, Mexico = undescribed species].-Rioja 1941: 680 (Mexican Pacific), 1962: 141 (Mexican Pacific).-Fauchald and Reimer 1975: 80 (Panama).-Fauchald 1977: 6 (Pacific Panama).-Van der Heiden and Hendrickx 1982 (Mexican Pacific).-Blake 1991 (Galapagos).-Hernández-Alcantara et al. 2003: 6.-Salazar-Vallejo and Londoño-Mesa 2004: 49.-Salazar-Silva 2006: 151.

##### Holotype.

USNM 19400.

##### Type locality.

Chatham Island, Galapagos Archipelago

##### Distribution.

Eastern Pacific from Mexico to Galapagos.

#### 13. Lepidonotus panamensis

Taxon classificationAnimaliaPhyllodocidaPolynoidae

Hartman, 1939

Lepidonotus pomareae panamensis Hartman, 1939: 44–46, pl. 6: figs 70–77 (Pacific Panama, 27–91m).
Lepidonotus panamensis .-Hartman 1959: 90.-Rullier and Amoureux 1979: 152 (Bahia).-Amaral and Nonato 1982: 25 (Espírito Santo).

##### Holotype.

USNM 47981.

##### Type locality.

Pacific, Panama.

##### Distribution.

Eastern Pacific: Panama Bay. Southwestern Atlantic from Bahia and Espírito Santo. Brazil, 27–91 m.

#### 14. Lepidonotus savignyi

Taxon classificationAnimaliaPhyllodocidaPolynoidae

Grube, 1856

Polynoe Grube, 1856: 45 (Callao, Peru); 1876: 61.-Seidler 1924: 88.
Lepidonotus savignyi .-Baird 1865: 183.-Augener 1925 (West Indies).-Salazar-Vallejo and Eibye-Jacobsen 2012: 1398.
Polynoe .-Quatrefages 1866: 225.

##### Type locality.

Callao, Peru.

##### Distribution.

Eastern Pacific from Peru. Western Atlantic from the West Indies.

#### 15. Lepidonotus sublevis

Taxon classificationAnimaliaPhyllodocidaPolynoidae

Verrill, 1873

Lepidonotus sublevis -Verrill 1973.-Verrill and Smith 1874: 581, pl. 10: fig. 42 (Savin Rock, near New Haven, and Vineyard Sound, Massachusetts); Verrill 1882: 300, pl. 4: fig. 2; pl. 6: fig. 3 (New England).-Dorner 1877: 66 (Massachusetts).-Wood 1885: 532 (New England).-Heilprin 1888 [1906]: 6, pl. 8: fig. 8 (New Jersey).-Andrews 1891: 278 (North Carolina).-Drowne 1896: 74 (Massachusetts).-Summer et al. 1913: 618 (Massachusetts).-Pratt 1923: 285.-Hartman 1942: 22–23, figs 7–12, 1944; 1945: 10 (North Carolina); 1951: 17–18 (Gulf of Mexico), 1954: 413 (Gulf of Mexico).-Ferguson and Jones 1949 (Virginia).-Behre 1950: 11 (Louisiana).-Hedgpeth 1950: 75 (Texas; on lower surface of *Renilla muelleri* [as *Renilla mülleri*]).-Pratt 1951: 328.-Wells 1961: 247 (North Carolina).-Pettibone 1963: 18–19, fig. 3e (Massachusetts to Florida, in gastropod shells with *Pagurus pollicaris*).-Wells and Gray 1964 (North Carolina).-Smith 1964: 76 (Massachusetts).-Yentsch et al. 1966: 117 (Massachusetts).-Calder and Brehmer 1967 (Virginia).-Conner et al. 1972: 1503.-Boesch 1973: 230 (Virginia).-Day 1973: 6 (Beaufort, North Carolina, 0–100 m).-Fotheringham 1976: 574 (Texas; comensal with *Clibanarius vittatus* and *Pagurus pollicaris*).-Gardiner 1976: 86, fig. 1f–g (North Carolina; associated with *Clibanarius vittatus*, *Pagurus impressus*, *Pagurus longicarpus*, and *Pagurus pollicaris*).-Kinner and Maurer 1978: 217 (Delaware Bay).-Dueñas 1981 (Caribbean Colombia), 1999 (Caribbean Colombia).-Stainken 1984: 100 (New York).-Weston 1984: 27 (Gulf of Mexico).-Ismail 1985: 384 (New Jersey).-Ibarzabal 1986: 2 (Cuba, 2 m); 1989b: 2–3, figs. 1a–f (Cuba).-Steimle and Caracciolo-Ward 1989: 148 (New York).-Dauer 1991 (comensal of *Clibanarius vittatus*, *Pagurus annulipes*, *Pagurus longicarpus*, and *Pagurus pollicaris*).-Bastida-Zavala 1995: 14 (Pacific Mexico).-Pollock 1998: 197 (northeast North America).-Posey et al. 1998: 151 (Gulf of Mexico).-Sagasti et al. 2000: 481 (Chesapeake Bay).-McDermott 2001: 624 (New Jersey; obligate symbiont of *Pagurus longicarpus*).-Baéz and Ardilla 2003: 102 (Caribbean Colombia, to 10 m).-Williams 2003: 17 (commensal of *Pagurus longicarpus*).-Williams and McDermott 2004: 28.-Struck et al. 2008: 631.-Fauchald et al. 2009: 768 (Gulf of Mexico).-Gobin 2010: 151 (Trinidad and Tobago).-Struck and Halanych 2010: 271.-Piquet et al. 2011: 417 (Antarctica).-Golombek et al. 2013: 315.
Lepidonotus sublaevis .-Seidler 1924: 41.-Gambi et al. 1997: 1055.
Lepidonotus pallidus Treadwell, 1939a: 3, figs 10–12 (Texas).
Lepidonotus squamatus .-Warren 1942: 45 (Louisiana).-Cowles 1930: 341.
[not Lepidonotus squamatus (Linnaeus, 1758); [= *Lepidonopsis humilis* (Augener 1922)].

##### Holotype.

PMNH 3-5.

##### Type locality.

New England (Northeastern USA).

##### Distribution.

Western Atlantic from Massachusetts to Colombia, with one record for Antarctica. Eastern Pacific from Mexico. From 2 to10 m.

##### Biology.

This species is commensal with the hermit crabs *Clibanarius vittatus* (Bosc, 1802), *Pagurus annulipes* (Stimpson, 1860), *Pagurus impressus* (Benedict, 1892) *Pagurus longicarpus* Say, 1818, and *Pagurus pollicaris* Say, 1818. It was found on the lower surface of the sea pansy, *Renilla muelleri* Kölliker, 1872 (Martin and Britayev 1998).

#### 16. Lepidonotus tenuisetosus

Taxon classificationAnimaliaPhyllodocidaPolynoidae

(Gravier, 1902)

Euphione tenuisetosa Gravier, 1902: 222–226, figs 228–231, pl. 8: figs 123–125 (Djibouti, Gulf of Tadjoura, Gulf of Aden).-Fauvel 1911: 368 (Persian Gulf).-Solís-Weiss et al. 2004: 13.
Lepidonotus tenuisetosus .-Fauvel 1919a: 330–332 (Madagascar), 1927: 411 (Suez Canal); 1933: 15 (India); 1953: 36–37, fig. 14c–f (India).-Seidler 1924: 25–27 (Red Sea).-Day 1934: 20 (Madagascar); 1953: 400 (South Africa); 1962: 632 (Madagascar); 1967: 82, fig. 1.14a–e (South Africa, Mozambique, Madagascar, and Red Sea); 1974 (Mozambique).-Monro 1934: 358 (China).-Aziz 1938 (Pakistan).-Okuda 1940: 4–6, fig. 2 (Japan).-Day and Morgans 1956 (South Africa).-Kalk 1958: 232 (Mozambique).-Macnae and Kalk 1958 (Mozambique).-Imajima and Hartman 1964: 27 (Japan).-Tampi and Rangarajan 1964: 100 (Andaman Islands).-Wu 1968: 27–28 (China).-Achari 1969: 31 (Andaman Islands).-Mohammad 1971: 288 (Kuwait).-Ben-Eliahu 1972: 190, 195 (Suez Canal).-Santhakumari 1973: 179 (India).-Hartman 1974 (Indian Ocean).-Peyrot-Clausade 1974 (Australia).-Sarma 1974: 158 (India).-Rullier and Amoureux 1979: 152 (Bahia).-Buzhinskaya et al. 1980: 228 (Indo-Pacific).-Soota et al. 1980 (Andaman and Nicobar Islands), 1981 (India).-Amaral and Nonato 1982: 25 (Espírito Santo).-Shin 1982 (China), 1998 (China), 2000 (China).-Uschakov 1982: 106, 107, pl. 29: figs 1–8 (Russia).-Kirkegaard 1983: 194, fig. 1.14a–e (Sierra Leone and French Guinea, 15–65 m).-Ansari et al. 1986: 363 (India).-Cantone 1987: 75, 80 (Somalia).-Gil et al. 1987: 1 (Korea).-Srikrishnadhas et al. 1987 (India).-Palpal-Latoc 1990 (Philippines); 1994: 67 (Philippines).-Hanley 1992: 366–367 (China, 0–0.5 m).-Hong and San 1993 (Vietnam).-Wang and Huang 1994: 4 (China).-Misra 1995: 93 (India).-Mustaquim 1997: 221 (Pakistan).-Wu et al. 1997 (China).-Che et al. 1999 (China).-Kumar 2000: 441 (India).-Misra and Chakraborty 2000: 219 (India).-Paxton and Chou 2000: 210 (China).-Bellan 2001: 224 (Europe).-Pillai 2001: 122 (India).-Sato 2001 (Japan).-Wehe and Fiege 2002: 113 (Arabian Peninsula).-Barnich and Fiege 2003: 86, fig. 44 (Mediterranean Sea).-Rasheed and Mustaquim 2003: 70–72, fig. 12 (Pakistan).-Barnich et al. 2004: 300–301 (China).-Khan and Murugesan 2005: 116 (India).-Zenetos et al. 2005: 73 (casual alien in Mediterranean), 2010 (introduced into Mediterranean).-Galil 2006 (Suez Canal), 2007: 301 (alien in Israel).-Kato et al. 2006: 30 (Japan).-Paiva 2006: 268 (Central Brazilian Plataform).-Wehe 2006: 107–109, fig. 24a–l (Arabian Peninsula).-Pleijel 2007: 179 (New Caledonia).-Li and Ping 2008 (China).-Çinar 2009: 2286, fig. 2a (Turkish Mediterranean), 2013: 1259 (introduced from Red Sea to Mediterranean).-Naeini and Rahimian 2009: 59–60 (Persian Gulf and Gulf of Oman).-Sukumaran and Devi 2009: 1443 (India).-Li et al. 2010: 110 (China).-Amaral and Nallin 2011: 556 (São Paulo).-Çinar 2013: 264 (alien species in Turkey).-Rizzo et al. 2011: 133 (São Paulo).-Wang 2011: 746 (China).-Amaral et al. 2013: 453 (São Paulo).-Rajasekaran and Fernando 2012: 3 (Andaman and Nicobar Islands).-Katsanevakis et al. 2012: (alien species in European waters).-Kazmi and Naushaba 2013: 253 (Pakistan).
Lepidonotus natalensis Day, 1951: 9, fig. 1e–l (Port Edward, Natal, South Africa).-Wehe 2006: 101–103, fig. 21a–j (Arabian Peninsula).-Naeini and Rahimian 2009: 55–59 (Gulf of Oman).
Lepidonotus cf. tenuisetosus .-Yan and Huang 1993: 133 (China).

##### Holotype of *Euphione tenuisetosa*.

MNHN Poly type 263.

##### Type locality.

Djibouti, Gulf of Tadjoura, Gulf of Aden.

##### Distribution.

Southwestern Atlantic from Bahia to São Paulo. Eastern Atlantic from Mediterranean to South Africa. Indian Ocean, Madagascar, Persian Gulf, and Red Sea. Western Pacific from Russia to Australia. 0–0.5 m.

##### Remarks.

Zenetos et al. (2010) and Çinar (2013) assigned *Lepidonotus tenuisetosus* as an exotic species for the Mediterranean Sea, with an origin in the Indo-Pacific/Red Sea. Its establishment success is uncertain, because it was recorded only once. We consider this species as an exotic species for the Brazilian coast.

#### 17. Lepidonotus tomentosus

Taxon classificationAnimaliaPhyllodocidaPolynoidae

(Grube, 1856)

Polynoe tomentosa Grube, 1856 (Punta Arenas, Chile).-Quatrefages 1866: 225–226.
Polynoe (Lepidonotus) pilosella Grube, 1878.
Lepidonotus tomentosus .-Fauvel 1919b: 472–473, fig. 1a–d (French Guyana), 1923 (French Guyana).-Hartman 1959.-Perkins and Savage 1975: 21.-Dean 2004 (Costa Rica).

##### Type locality.

Punta Arenas, Chile.

##### Distribution.

Eastern Pacific from Costa Rica and Chile. Western Atlantic from French Guyana.

##### Remarks.

There is material in the USNM from the Galapagos Islands.

#### 18. Lepidonotus variabilis

Taxon classificationAnimaliaPhyllodocidaPolynoidae

Webster, 1879

Lepidonotus variabilis Webster, 1879: 205–208, pl. 1: figs 6–11; pl. 2: figs 12–14 (Virginia).-Andrews 1891: 278 (North Carolina; among hydroids and sponges).-Hoagland 1919: 572 (Puerto Rico, 18 m).-Seidler 1924: 70–72.-Treadwell 1939b: 185 (Puerto Rico).-Warren 1942: 45 (Louisiana).-Hartman 1945: 10 (Florida), 1951: 18 (Gulf of Mexico), 1954: 413–414 (Gulf of Mexico).-Behre 1950: 11 (Louisiana).-Pearse and Williams 1951 (North and South Carolina).-Renaud 1956: 3, fig. 2 (Bahamas).-Rioja 1958: 221 (eastern Mexico).-Wells 1961: 247 (North Carolina).-Wells and Gray 1964 (North Carolina).-Ebbs 1966: 493–496, fig. 2a–h (Florida).-Forbes 1966: 278 (Florida; associated with Cryptostrea [as Ostrea] permollis and Stelletta grubii).-Dauer 1973: 193 (Gulf of Mexico; in sponge).-Day 1973: 6 (North Carolina, to 18 m).-Rullier 1974: 20 (Cuba; in sponges).-Gardiner 1976: 86, fig. 1k–n (North Carolina).-MacPhee 1978: 15 (Massachusetts; food of Pseudopleuronectes americanus).-Rodríguez-Gómes 1979: 27 (Caribbean Colombia); 1988 (Caribbean Colombia).-Tagatz et al. 1982: 134 (Alabama).-Ibarzábal 1986: 2 (Cuba, 3 m).-San Martín et al. 1986: 6–7 (Cuba).-Perkins 1998: 85 (Florida).-Dueñas 1999 (Caribbean Colombian).-Baéz and Ardilla 2003: 102 (Caribbean Colombian, 0.2–2 m).-Fauchald et al. 2009: 768 (Gulf of Mexico).-Gobin 2010: 5 (Trinidad and Tobago).
not Lepidonotus variabilis .-Treadwell 1939b: 341. [=Lepidonotus subleavis Verrill, 1874]

##### Type locality.

Virginia Coast, North American.

##### Distribution.

Western Atlantic from Massachusetts to Colombia; 0.2–18 m.

##### Biology.

Associated with the oyster *Cryptostrea permollis* (Sowerby, 1871), and with the sponge *Stelletta grubii* Schmidt, 1862. Food of the winter flounder, *Pseudopleuronectes americanus* (Walbaum, 1792). Lives among hydroids and sponges.

## Discussion

Herein, all information on members of the genus *Lepidonotus* found around South American coasts in the literature have been gathered, and additional data on two species collected in northeastern Brazil is provided. Eighteen species and subspecies are catalogued from South America, and three of them represent endemic taxa: *Lepidonotus brasiliensis* and the subspecies *Lepidonotus brasiliensis brevis* are endemic for Bahia, Brazil, while *Lepidonotus margaritaceus* is endemic from Ecuador. The subspecies *Lepidonotus brasiliensis brevis* is very similar to the species *Lepidonotus brasiliensis*. However, only a detailed review can confirm if the two taxa are synonyms.

The species *Lepidonotus caeruleus*, *Lepidonotus carinulatus*, *Lepidonotus hupferi*, *Lepidonotus natalensis*, ﻿and *Lepidonotus tenuisetosus* have a broad distribution, and have been reported from several countries. According to Zenetos et al. (2010) and Çinar (2013), the species *Lepidonotus carinulatus* and *Lepidonotus tenuisetosus* are exotic species in the Mediterranean and have possibly originated in the Indo-Pacific region/Red Sea. Their introduction area was through the Mediterranean and Sea of Marmara. Despite the broad distributions of *Lepidonotus caeruleus*, *Lepidonotus hupferi* and *Lepidonotus natalensis*, ﻿more studies are needed to indicate if they may possibly represent exotic species, their possible areas of introduction, and into which ecological category they belong according to the classification scheme of Çinar (2013). The possible origin of *Lepidonotus natalensis* is Natal, South Africa, and it was possibly reported from the Suez Canal, Arabian Sea, and Karachi, Pakistan. It is herein reported from the southwest Atlantic, in the state of Paraíba. *Lepidonotus caeruleus* was first described off Rio de Janeiro, and was reported from the Pacific coast of North America and Japan. *Lepidonotus hupferi* was first described from the Eastern Atlantic from Senegal and Cape Verde, and later reported for the Pacific from New Caledonia, Australia, Mexico, and Ecuador. The remaining species present narrow distributions spanning few countries.

For some records essential features are not presented clearly, such as the ornamentation of the elytra, or the shape of the prostomium and chaetae. Some characters, such as form of nuchal folds, pigmentation of the antennae, and dorsal cirri, are not mentioned for the species *Lepidonotus brasiliensis* and *Lepidonotus panamensis*. We are left with the view that species are very similar and difficult to distinguish. Therefore, revisionary studies of *Lepidonotus* are needed to establish whether cryptic species occur.

## Supplementary Material

XML Treatment for Lepidonotus

XML Treatment for Lepidonotus arenosus

XML Treatment for Lepidonotus brasiliensis

XML Treatment for Lepidonotus brasiliensis laevis

XML Treatment for Lepidonotus caeruleus

XML Treatment for Lepidonotus carinulatus

XML Treatment for Lepidonotus crosslandi

XML Treatment for Lepidonotus crosslandi peruana

XML Treatment for Lepidonotus furcillatus

XML Treatment for Lepidonotus hupferi

XML Treatment for Lepidonotus margaritaceus

XML Treatment for Lepidonotus natalensis

XML Treatment for Lepidonotus nesophilus

XML Treatment for Lepidonotus panamensis

XML Treatment for Lepidonotus savignyi

XML Treatment for Lepidonotus sublevis

XML Treatment for Lepidonotus tenuisetosus

XML Treatment for Lepidonotus tomentosus

XML Treatment for Lepidonotus variabilis
